# Reactions of *N*-heterocyclic carbenes with nitrobenzenes: harnessing NHCs as synthetic building blocks

**DOI:** 10.1016/j.jare.2025.11.036

**Published:** 2025-11-19

**Authors:** Zhiguo Zheng, Youlin Deng, Xiaoqun Yang, Zhichao Jin, Shiqing Huang, Jian Wu, Yonggui Robin Chi

**Affiliations:** a State Key Laboratory of Green Pesticide, Guizhou University, Guiyang 550025, China; b School of Chemistry and Life Resources, Renmin University of China, Beijing 100872, China; c School of Chemistry, Chemical Engineering, and Biotechnology, Nanyang Technological University, Singapore 637371 Singapore

**Keywords:** Nitrobenzene, *N*-heterocyclic carbene, Cascade reaction, Quinazolin-4-one derivatives, Antifungal activity

## Abstract

**Introduction:**

Quinazolin-4-one is a privileged scaffold in drugs with broad clinical applications. Conventionally, an *o*-amino group was generally needed to be pre-installed on the benzoic acid-derived starting materials for the efficient condensation reactions towards quinazolin-4-ones. The application of benzene derivatives without requisite amino and / or carbonyl groups as the quinazolin-4-one starting materials has not been disclosed. Therefore, we report herein the fabrication of NHC molecules with nitrobenzenes for facile access to quinazolin-4-one derivatives.

**Objectives:**

To develop an unprecedented synthetic method utilizing nitrobenzenes and *N*-heterocyclic carbene (NHC) molecules, to elucidate the novel reaction mechanism through experimental and computational studies, to synthesize a series of quinazolin-4-one derivatives, and to evaluate their in vitro antibacterial activity.

**Methods:**

A synthetic method involving nitrobenzenes and NHC molecules was developed. The novel reaction mechanism was elucidated through isotopic labeling experiments and computational studies, and a series of quinazolin-4-one derivatives were synthesized. The synthesized compounds were evaluated for in vitro antifungal activity against four plant pathogens using the mycelial growth rate method.

**Results:**

We report for the first time an unprecedented synthetic method involving nitrobenzene and NHC molecules. The nitrobenzene substrate acts as a novel type of NHC acceptor, enabling a condensation reaction with the NHC molecule through a multi-step nucleophilic addition/rearrangement/dehydration cascade process. This conceptually novel reaction mechanism has been thoroughly investigated through experimental and computational studies. A variety of substituents with different electronic properties are tolerated on both the nitrobenzene and NHC reactants. A series of quinazolin-4-one products can be readily accessed using the current synthetic strategy, and they exhibit promising antifungal activity in plant protection.

**Conclusion:**

We have developed a conceptually novel incorporation reaction between nitrobenzenes and NHC molecules. Synthesized compound 13 exhibited broad-spectrum antifungal activity, providing a basis for its development potential as a new framework for pesticide design.

## Highlights

• We have developed a conceptually novel incorporation reaction between nitrobenzenes and NHC molecules.
• Through experimental and computational studies, an in-depth investigation of the reaction mechanism was conducted, revealing an unprecedented reaction pathway involving the NHC structure.
• The nitrobenzene substrate and the NHC framework both tolerate a variety of substituents well, leading to the generation of a range of quinazolin-4-one products.
• The obtained quinazolin-4-one product 13 exhibits good bioactivity against plant fungi, highlighting their potential in crop protection.

## Graphical abstract

**Figure:**
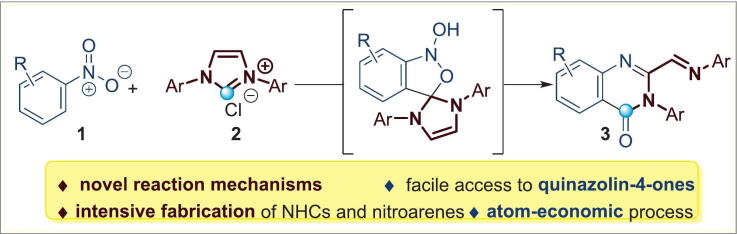

## Introduction

**Fig. 1:**
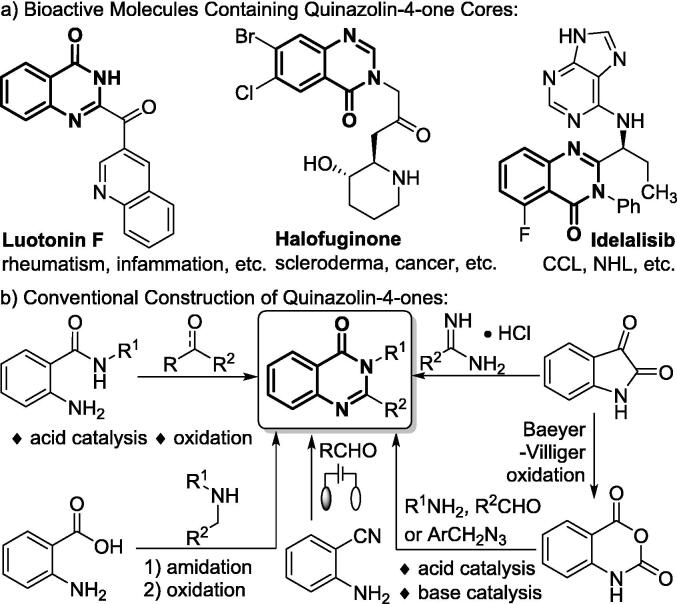
Quinazolin-4-ones in bioactive molecules and their syntheses.

Quinazolin-4-one is a privilege structural core in human medicines that have been adopted in clinical treatment of many diseases (Fig. 1a) [[1], [2], [3], [4], [5], [6], [7], [8], [9], [10], [11], [12], [13], [14], [15]]. For instance, Luotonin F [[6], [7], [8]] is a naturally occurred bioactive molecule inside the Chinese medicine of *Peganumnigellastrum Bunge*, which has long been used in the treatment of rheumatism, inflammation, and abscesses. Halofuginone [[9], [10], [11], [12]] is a synthetic drug bearing a substituted quinazolin-4-one core and has been used for the treatment of scleroderma, cancer, and restenosis. Idelalisib [[13], [14], [15]] is the principal drug in the clinical treatment of relapsed chronic lymphocytic leukaemia (CLL), follicular B-cell non-Hodgkin’s lymphoma (NHL) and small lymphocytic lymphoma (SLL). Therefore, it is significant and practically useful to develop facile approaches to construct quinazolin-4-one derivatives.

Conventionally, the quinazolin-4-one could be formed from 2-aminobenzamides through acid-promoted dehydrative cycloaddition reactions with the reagents / oxidated intermediates bearing carbonyl groups (Fig. 1b, top left) [[16], [17], [18], [19], [20], [21], [22], [23], [24], [25]]. They could also be approached from 2-aminobenzoic acids through an amidation / oxidation cascade process with secondary amines (Fig. 1b, bottom left) [[26], [27], [28]]. The 2-aminobenzonitrile could go through a reduction / condensation process with aldehydes under electrochemical conditions to give the target quinazolin-4-ones (Fig. 1b, bottom middle) [29]. Isatoic anhydride, which could be obtained from isatins through Baeyer-Villiger oxidation reactions, could also be used as the starting materials for the preparation of quinazolin-4-ones, with amines, azides or acetimidamides used as the nitrogen sources (Fig. 1b, right) [[30], [31], [32], [33], [34], [35], [36], [37], [38]]. It is perceived that an *o*-amino group was generally needed to be pre-installed on the benzoic acid-derived starting materials for the efficient condensation reactions towards the target quinazolin-4-one products. The application of benzene derivatives without requisite amino and / or carbonyl groups as the quinazolin-4-one starting materials has not been disclosed.

NHCs have nowadays been widely used as efficient organic catalysts and robust ligands in synthetic chemistry, including some of our work [[39], [40], [41], [42], [43], [44], [45], [46]]. They can activate various electron-deficient substrates to promote addition reactions in enantioselective or non-chiral fashion. The electronic and steric properties of the transition-metal catalysts could also be adjusted by the NHC ligands to help accelerate and control diverse cross-coupling and metathesis reactions. In contrast, the applications of NHCs as the reaction partners have rarely been reported [[47], [48], [49], [50], [51], [52], [53]]. Challenges might exist in the instabilities of the highly active and complex intermediates formed between the NHC molecules and the reaction substrates.

**Fig. 2:**
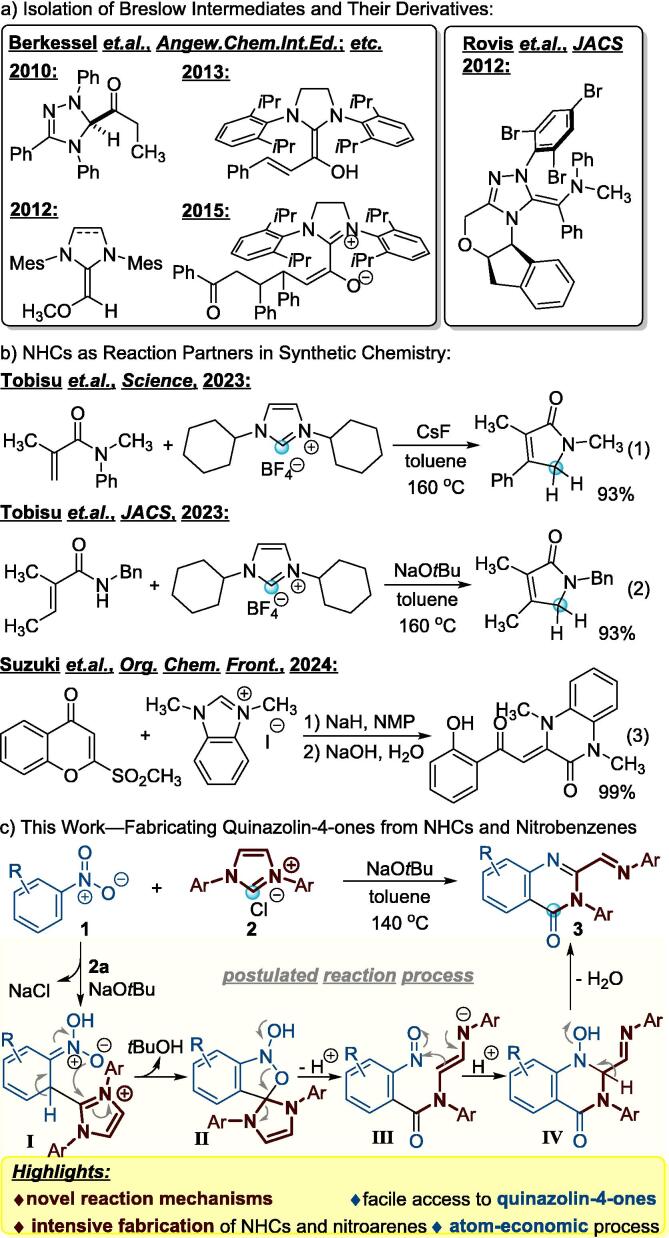
NHCs as reactants in organic synthesis.

To date, limited success has been achieved in the fabrication of NHC molecules into electron-deficient reactants (Fig. 2) [[47], [48], [49], [50], [51], [52], [53]]. For instance, Berkessel and co-workers have intensively explored the NHC-related intermediates throughout the NHC organocatalytic reaction processes and successfully isolated the Breslow intermediates, azolium enolate intermediates and their derivatives for mechanistic studies (Fig. 2a, left side) [[47], [48], [49], [50]]. Rovis and co-workers demonstrated that some aza-Breslow intermediates could be crystallized, fully characterized and adopted as the NHC catalyst-precursor in the intramolecular Stetter reaction (Fig. 2a, right side) [51]. Recently, Tobisu and co-workers disclosed that the imidazolium-derived NHCs could be adopted as the one-carbon synthon to furnish the single-carbon atom doping (SCAD) reaction with *α, β-*unsaturated amide substrates (Fig. 2b) [52,53]. With the *α, β-*unsaturated amide bearing an *N*-phenyl group used as the substrate, a single carbon from the NHC molecule could be added onto the electron-deficient system to bridge the alkene group and the amide nitrogen, with the *N*-phenyl group migrated to alkene moiety *via* a S_N_Ar process (eq. 1). When the amide contained a free N–H group, the direct doping of a single carbon atom produced the γ-lactam product in an excellent yield (eq. 2). Very recently, Suzuki and co-workers disclosed the carbon atom insertion reaction into the benzimidazolium salt-derived NHCs through a cascade addition / elimination / hydroxylation / ring-expansion / ring-opening process, with the 3,4-dihydroquinoxalin-2(1*H*)-ones afforded in up to 99 % yield (eq. 3) [54]. In addition, the inactivation of NHC catalysts sometimes resulted in the formation of unexpected structures that were consisted with the fragments from both the NHC catalyst and the reaction substrates, as disclosed by the groups of Rovis, Chi and others [[47], [48], [49], [50], [51],^55^]. The reported achievements generally required electron-deficient carbonyls, alkenes or imines as the NHC acceptors. To the best of our knowledge, the incorporation of NHC molecules into substituted benzene structures has not been successful.

The nitro group could significantly decrease the electron-density of the benzene ring, which could help increase the electrophilicity of the nitrobenzene toward the nucleophilic attack by an NHC. Therefore, we report herein the fabrication of NHC molecules with nitrobenzenes for facile access to quinazolin-4-one derivatives (Fig. 2c). The nitrobenzene substrate **1** undergoes nucleophilic attack by the free NHC molecule generated *in situ* from imidazolium salt **2** at the ortho-position, forming the dearomatized intermediate **I**. In distinction to the hydroxide addition process that reported by Suzuki and co-workers with the benzimidazolium salt-derived NHCs [54], the adduct **I** subsequently cyclizes to generate the spirocyclic intermediate **II** and goes through deprotonation by the tert*-*butoxide anion to drop one equivalent of tert*-*butanol. Intermediate **II** could be deprotonated by base and cleavage into the nitrosobenzene intermediate **III**. The nitrogen atom of the nitroso group in **III** is subjected to nucleophilic attack by the enamine moiety, leading to the formation of a six membered intermediate **IV**. This intermediate subsequently undergoes dehydration to ultimately produce the target quinazolin-4-one product **3**.

It is worth noting that nitrobenzenes have been studied as effective electrophiles in the NHC-catalyzed S_N_Ar reactions with various aldehydes [56]. In stark contrast to our work, the NHCs reacted as the reaction catalysts and were not incorporated into the aryl rings of the nitrobenzene structures in the reported S_N_Ar reactions. The current work unprecedentedly fabricates the structures of the NHC and the nitrobenzene through a mechanistically complex while operationally simple reaction involving cascade addition, rearrangement and dehydration processes, with only one equivalent of water molecule is lost throughout this reaction. In addition, the NHC-nitrobenzene chemistry could give the target quinazolin-4-one products in a facile manner without the use of any heavy metal. It could completely avoid the trans-metal contamination to the environment, which was inevitable in the traditional metal-catalyzed oxidation / reduction process in the quinazolin-4-one synthesis. The conceptually novel reaction mechanism for the complex incorporation strategy could be supported by both the control experiments and the DFT calculations.

## Materials and methods

### Materials and instruments

Water-18 (98 % ^18^O) was purchased from Energy chemical and used as received. Compound 1,3-Bis(2,6-diisopropylphenyl)imidazolium chloride (CAS: 250285–32-6) was purchased from Bide Pharmatech with a purity of 99.30 %, all nitrobenzene compounds were obtained from damas-beta with purities of 98 % or higher.

NMR spectra were recorded on Brüker ASCEND 400 spectrometers (400 MHz for ^1^H NMR, 101 MHz for ^13^C, and 377 MHz for ^19^F). Chemical shift (δ) for each ^1^H and ^13^C NMR spectrum is given in ppm, with the TMS used as the inner standard. The residual solvent signals of CHCl_3_ or DMSO were used as references for ^1^H and ^13^C NMR spectra (CDCl_3_: δ_H_ = 7.26 ppm, δ_C_ = 77.16 ppm). The following abbreviations were used to explain the multiplicities: s = singlet, d = doublet, t = triplet, q = quartet, m = multiplet. All first-order splitting patterns were assigned on the base of the appearance of the multiplet. Splitting patterns that could not be easily interpreted is designated as multiplet (m). High resolution mass spectrometer analysis (HRMS) was performed on Thermo Fisher Q Exactive mass spectrometer. Melting points were measured on a Beijing Tech Instrument X-4 digital display micro melting point apparatus and were uncorrected. Analytical thin-layer chromatography (TLC) was carried out on pre-coated silica gel plate (0.2 mm thickness), with visualization performed using a UV lamp.

### General procedure for the preparation of 3/4

Nitrobenzene **1** (0.10 mmol), NHC **2** (0.15 mmol), NaO*t*Bu (0.15 mmol) and toluene (1.0 mL) were added to a 10 mL screw-capped pressure-proof vial, and the resulting mixture was stirred at 140 °C using an aluminum block for 18 h followed by cooling to rt. The mixture was purified by column chromatography (PE / EtOAc = 100 / 1 to 50 / 1) to give the product **3**/**4**.

### In vitro antifungal activity assay

An in vitro antifungal activity test of four phytopathogens was conducted through the mycelial growth rate method. The phytopathogens include four fungi, *Rhizoctonia solani* (*Rs*), *Phomopsis* sp. (*Ps*), *Colletotrichum capsici* (*Cc*) and *Sclerotinia sclerotiorum* (*Ss*).

Dissolving 3.5 mg of target compounds in 350 μL of dimethyl sulfoxide (DMSO) produced a mother liquor with a concentration of 10 000 μg/mL. This mother liquor was then introduced into a 70 mL potato dextrose agar (PDA) medium to attain a test concentration of 50 μg/mL, which was subsequently poured into a 60 mm diameter Petri dish. Each treatment was replicated 3 times. Commercial fungicides azoxystrobin was employed as positive controls. The fungi in the Petri dish underwent incubation in a biochemical incubator at 28 ± 1 °C for 2–6 days. Once the mycelium without the addition of any fungicidal chemicals (CK) reached 2/3 of the Petri dish, the colony diameter was measured using the cross method. The inhibition rate is calculated as follow:

inhibitionrate=[(C-T)/(C-0.5)]∗100%

where C is the DMSO-treated colony diameter, T is the compound-treated colony diameter, and 0.5 is the diameter of mycelium cakes (cm).

## Results and discussion

### Reaction development

We initially tested the feasibilities of the incorporation processes between the nitrobenzene substrates **1** and various NHC precursors **2** in presence of the stoichiometric amount of the base NaO*t*Bu (Table 1, entries 1 to 5). The nitrobenzene **1a** could react with the NHC **2a** to give a trace amount of the target product **3a** (Entry 1). Introducing an electron-donating 2-methyl group onto the nitrobenzene structure (to afford **1b**) also yielded only a trace amount of the desired product **3b** (Entry 2). In contrast, the nitrobenzene substrate **1c** bearing an electron-withdrawing 2-chloro group gave the quinazolin-4-one product **3c** in a promising 65 % yield (Entry 3). Further decreasing the electron-density of the nitrobenzene substrate by installing an additional nitro group (to afford **1d**) resulted in only a trace amount of the product (Entry 4, **3d**). Moving the 2-chloro group on the substrate **1c** to the 4-position (to afford **1e**) resulted in a significant decrease on the product yield (Entry 5, **3e**).

**Table 1:** Optimization of reaction conditions.^a^


Entry	1	2	Base	Solvent	3 (Yield %)^b^
1	**1a**	**2a**	NaO*t*Bu	toluene	**3a** (< 5)
2	**1b**	**2a**	NaO*t*Bu	toluene	**3b** (< 5)
3	**1c**	**2a**	NaO*t*Bu	toluene	**3c** (65)
4	**1d**	**2a**	NaO*t*Bu	toluene	**3d** (< 5)
5	**1e**	**2a**	NaO*t*Bu	toluene	**3e** (19)
6	**1c**	**2b**	NaO*t*Bu	toluene	0
7	**1c**	**2c**	NaO*t*Bu	toluene	0
8	**1c**	**2d**	NaO*t*Bu	toluene	0
9	**1c**	**2e**	NaO*t*Bu	toluene	< 5
10	**1c**	**2a**	Na_2_CO_3_	toluene	**3c** (52)
11	**1c**	**2a**	KO*t*Bu	toluene	**3c** (54)
12	**1c**	**2a**	Et_3_N	toluene	**3c** (0)
13	**1c**	**2a**	DBU	toluene	**3c** (11)
14	**1c**	**2a**	NaO*t*Bu	DMF	**3c** (< 5)
15	**1c**	**2a**	NaO*t*Bu	*n*BuOH	**3c** (0)
16	**1c**	**2a**	NaO*t*Bu	H_2_O	**3c** (0)
17	**1c**	**2a**	NaO*t*Bu	1,4-dioxane	**3c** (64)
18	**1c**	**2a**	NaO*t*Bu	mesitylene	**3c** (64)
19^c^	**1c**	**2a**	NaO*t*Bu	toluene	**3c** (72)
20^c^	**1c**	**2a**	NaO*t*Bu	toluene	**3c** (51^d^/70^e^)
21^c^	**1c**	**2f**, **2g**, **2h**, **2i**, **2j, 2k, 2l**	NaO*t*Bu	toluene	0

a Reaction conditions: 1a (0.1 mmol), 2 (0.1 mmol), base (0.1 mmol) and solvent (1.0 mL) at 140 °C for 18 h.

b Data in parentheses were isolated yields of 3 after column chromatography.

c 2a (0.15 mmol) and NaO*t*Bu (0.15 mmol) was used under otherwise the same condition.

d The reaction was carried out at 120 °C for 18 h.

e The reaction was carried out at 160 °C for 18 h.

Subsequently, we investigated the influence of the NHC substitution patterns on the formation of the quinazolin-4-one products (Entries 6 to 9). Replacing the *N*-aryl groups on the NHC precursor **2a** with alkyl groups (e.g., **2b** and **2c**) led to no reactions under the current condition, with the starting material **1c** recovered in good yields (Entries 6 to 7). The NHC precursor **2d** bearing electron-deficient *N*-(4-bromo)phenyl groups also did not work in this protocol (Entry 8). Switching the 2,6-diphenyl groups on the NHC structure into mesityl groups also resulted in little formation of the desired product (Entry 9).

Therefore, we continued to optimize the reaction condition using the nitrobenzene substrate **1c** and the NHC precursor **2a** (Entries 10 to 20). A diversity of inorganic bases could promote the fabrication between the nitrobenzene **1c** and the NHC **2a**, although the yields were relatively lower (52 % and 54 %) under the current condition (Entries 10 to 11). Organic bases such as Et_3_N and DBU were not efficient for this transformation, affording yields of 0 % and 11 %, respectively (Entries 12 to 13). Solvents with high polarities were not suitable for this reaction (Entries 14 to 16), while non-polar solvents could generally give 64 % yields of the final products (Entries 17 to 18). Increasing the amount of the NHC **2a** and the base NaO*t*Bu could promote the product yield to 72 % (Entry 19). Further decreasing or increasing the reaction temperature failed to improve the product yields (Entry 20). It is worth noting that the product **3c** have shown excellent stabilities in various solutions under different conditions at 140 °C, with the recovery yield ranging from 95 % to 99 % after stirring for 24 h (for details, see the Supporting Information on Pages 50 to 51). Changing the imidazolium-derived NHC **2c** into other types of classical NHCs, such as the triazliums (**2f**, **2 g**), thioazliums (**2 h**, **2i**) and the benzimidazole (**2j, 2 k, 2 l**), resulted in no formations of the desired fabrication products, with all the starting materials remained unchanged under the currently optimized reaction condition. Switching the NHCs into other organic bases such as the TEA and DMAP also resulted in no reaction. Both of the nucleophilic carbons and the 5-membered ring structures in NHC molecules are significant for the current fabrication reaction.

### Reaction scope

**Scheme 1:**
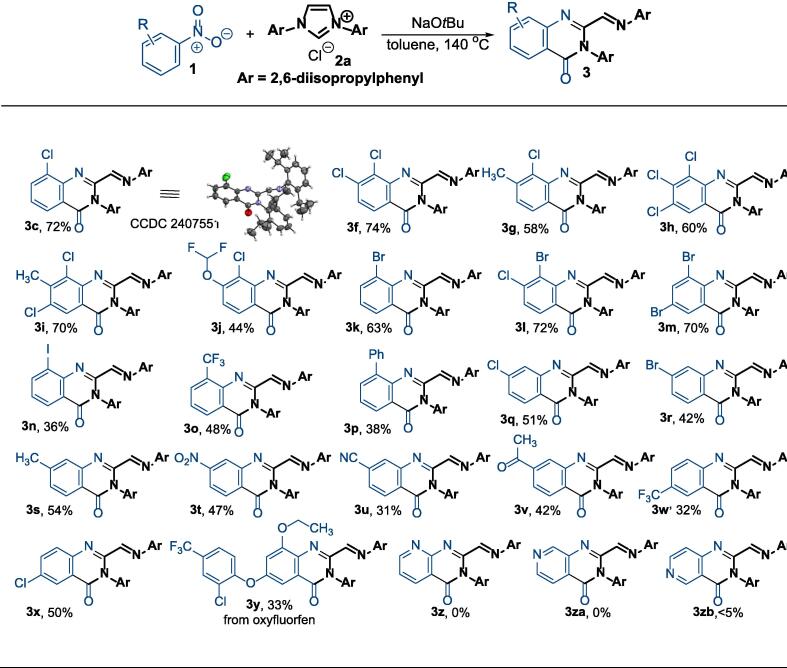
Scope of the nitrobenzene substrates 1.*^a a^*Reaction conditions: 1 (0.1 mmol), 2a (0.15 mmol), NaO*t*Bu (0.15 mmol) and toluene (1.0 mL) at 140 °C for 18 h. Yields are isolated yields after purification by column chromatography.

Having identified an optimal reaction condition for the fabrication of the nitrobenzene **1c** and the NHC **2a**, we next examined the functional group tolerance on the nitrobenzene substrate **1** (Scheme 1). Both electron-donating and electron-withdrawing groups could be well tolerated on the 2-chloronitrobenzene **1c**, with the corresponding products **3f** to **3j** afforded in 44 % to 74 % yields. The 2-chloro groups on the nitrobenzene substrates could be switched into 2-bromo groups without much erosion on the product yields (**3 k** to **3 m** with 63 % to 72 % yields). In contrast, switching the 2-chloro group on **1c** into a 2-iodo, 2-trifluoromethyl or 2-phenyl group resulted in significant decrease on the product yields (**3n** to **3p**). Moving the 2-substituents on the nitrobenzene substrates to the 3- or 4-position led to drops on the reaction yields, regardless of their electronic properties (**3q** to **3x**). It was pleasing to find that the herbicide of oxyfluorfen could be adopted as an effective substrate in the current transformation, although the fabricated product **3y** could only be achieved in a 33 % yield at the current stage. Switching the substituted phenyl group of the nitrobenzene substrate into various pyridinyl groups resulted in < 5 % yield for the formation of the target products (**3z**, **3za**, **3zb**).

**Scheme 2:**
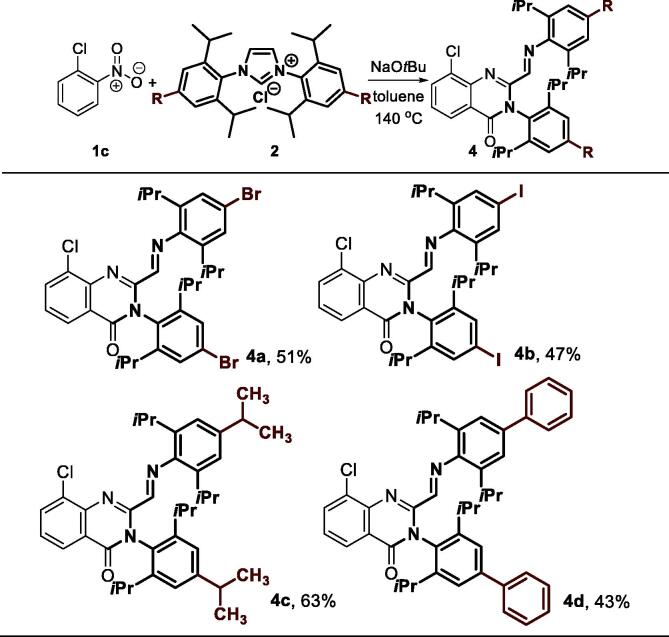
Scope of the NHCs 2.*^a a^*Reaction conditions: 1c (0.1 mmol), 2 (0.15 mmol), NaO*t*Bu (0.15 mmol) and toluene (1.0 mL) at 140 °C for 18 h. Yields are isolated yields after purification by column chromatography.

Consequently, the substituents on the NHC reaction partner were also investigated (Scheme 2). The 2,6-diisopropyl groups were found critical to the generation of the target products. Electron-withdrawing halo groups could be introduced onto the 4-position of the *N*-aryl moiety of the NHC to give the target products in moderate yields (**4a** to **4d**). An additional electron-donating 4-isopropyl group on the *N*-aryl moiety of **2a** led to a slight drop in the product yield. A phenyl substituent on the same position resulted in even lower reaction efficiency.

### Mechanistic studies

**Fig. 3:**
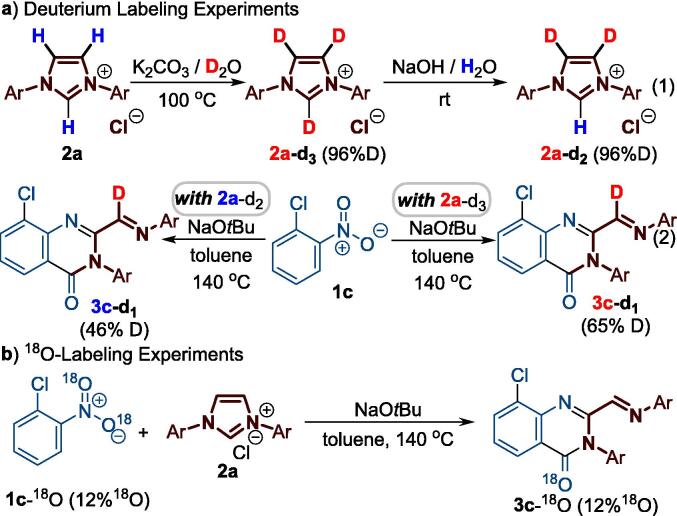
Isotope labelling experiments. Ar = 2,6-diisopropylphenyl. (a) Deuterium labeling experiments (b) ^18^O-labeling experiments.

To further investigate the reaction mechanism, we carried out control experiments using isotope labelled NHC substrates **2a** (Fig. 3). The fully deuterated **2a** could be obtained in 96 % yield after stirring with D_2_O at 100 °C for 24 h (Fig. 3a, eq. 1, **2a**-d_3_). Then the most acidic D^+^ on **2a**-d_3_ could be switched with H_2_O under basic condition to give the di-deuterated **2a** in 96 % yield (Fig. 3a, eq. 1, **2a**-d_2_). The mono-deuterated product **3c** could be obtained from the reactions between the 2-chloronitrobenzene **1c** and the deuterated NHC **2a** (Fig. 3a, eq. 2, **3c**-d_1_). When the fully deuterated **2a**-d_3_ was used as the reaction substrate, the **3c**-d_1_ was obtained with 65 % deuterated ratio. The deuterated ratio of the **3c**-d_1_ was reduced to 46 % when replacing the fully deuterated **2a**-d_3_ with di-deuterated **2a**-d_2_. The results indicated that the H atom at the imine moiety in the product structure came from the NHC molecule. The D-H exchange between the deuterated NHC and the moistures from either the dehydration process or the reaction system led to the decrease of the deuterium ratio in the target products.

We also carried out the dehydrative coupling reaction between the NHC **2a** and the ^18^O-labeled nitrobenzene **1c**-^18^O (Fig. 3b). The ^18^O ratios in both the reaction starting material and the product were the same, indicating clearly that the amide oxygen came from the nitro group on the nitrobenzene substrate.

**Fig. 4:**
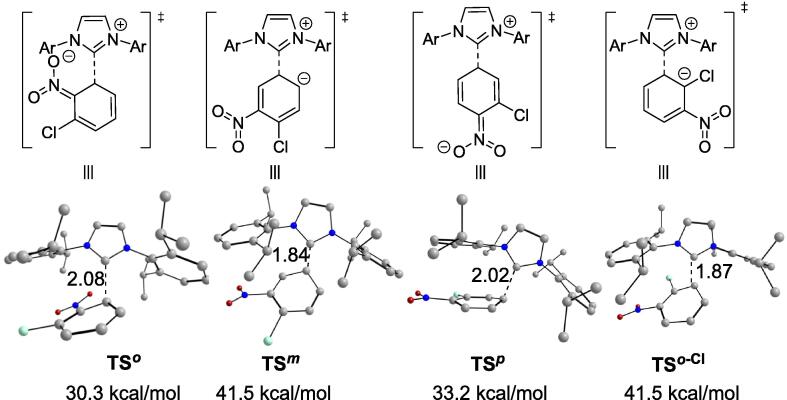
The calculated ΔG^≠^ as well as the optimized geometries of the corresponding transition states. Distances are given in Å.

In addition, we computed the activation free energies (ΔG^≠^) for the nucleophilic attack of the NHC **2a** on four different positions of the nitrobenzene **1c** (Fig. 4). The results indicate that the nucleophilic attack of the NHC is most favorable at the ortho-position to the nitro group (TS*^o^*, ΔG^≠^ = 30.3 kcal/mol). In contrast, the *meta*- and para-positions (TS*^m^* and TS*^p^*) have significantly higher activation barriers (41.5 and 33.2 kcal/mol, respectively), suggesting that these positions are less reactive. The high activation barrier for the attack at the ortho-position to the Cl group (TS*^o^*^-Cl^, 41.5 kcal/mol) indicates that Cl substitution adversely affects nucleophilicity at that site. This strongly supports the experimentally proposed mechanism. The selectivity toward the ortho-position to the nitro group may be attributed to the combined electronic effects of the nitro and the Cl substituents.

**Fig. 5:**
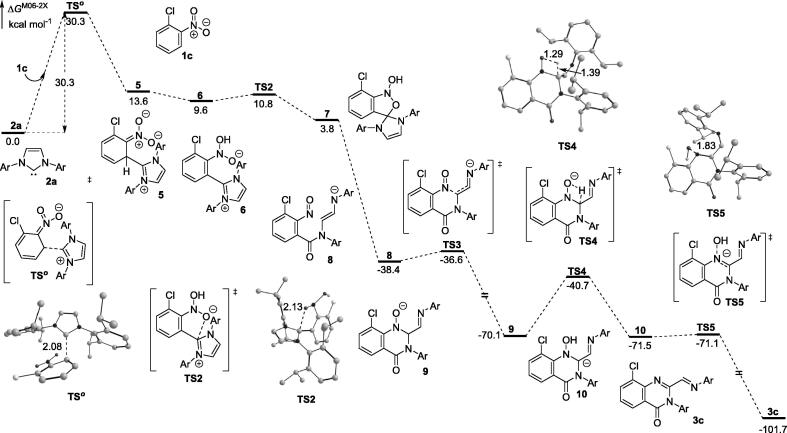
The energy profile of the proposed mechanism. Ar = 2,6-diisopropylphenyl.

The reaction mechanism was meticulously examined through density functional theory (DFT) calculations, focusing on the sequential transformations leading to the final product (Fig. 5). Detailed computational results and discussion on stereoselectivity are given in the Supplementary Information on Pages S21 to S48. The process begins with the nucleophilic attack of NHC **2a** at the ortho-position to the nitro group in the substrate **1c**. The computed Gibbs activation energy for this step is 30.3 kcal/mol, which is supposed to be overcome under the experimental conditions at 140 °C, rendering this step kinetically viable. This attack results in the formation of a dearomatized intermediate **5**, with a Gibbs free energy of 13.6 kcal/mol relative to the reactant state (**1c** + **2a**).

Subsequently, intermediate **5** undergoes a proton transfer, where one of the oxygen atoms in the nitro group receives a proton, thereby reinstating the aromaticity of the benzene ring. This transformation yields intermediate **6**, which is more stabilized with a Gibbs free energy of 9.6 kcal/mol. Following this, the negatively charged oxygen in the nitro group facilitates an intramolecular nucleophilic attack on the carbene carbon, forming a spirocyclic intermediate **7**. Notably, this step is associated with an exceptionally low energy barrier of only 1.2 kcal/mol, suggesting that the cyclization occurs with remarkable ease, likely due to the ionic nature of the reaction. The resultant intermediate **7** exhibits a Gibbs free energy of 3.8 kcal/mol.

Under basic reaction conditions, the hydroxyl proton in intermediate **7** is readily abstracted by the base, leading to the formation of intermediate **8**. This deprotonation step is highly exergonic, as evidenced by a Gibbs free energy of −38.4 kcal/mol. After that, the C=C bond of the enamine moiety in **8** acts as a nucleophile, attacking the nitrogen atom of the N=O moiety. Various methods (e.g., modifying the TS from the unsubstituted system followed by mod redundant job and TS search job; using QST2/3; relaxed PES scan then use the local maximum as guess structure etc) have been tried to locate this TS but to no avail. Consequently, the energy barrier was approximated by scanning the N-C distance and selecting the highest energy point along the scan. The estimated energy barrier for this step is merely 1.8 kcal/mol, though this may be an overestimation.

Following this step, a new N-C bond is formed, yielding intermediate **9**, which exhibits substantial thermodynamic stability, as reflected by a Gibbs free energy of −70.1 kcal/mol. This pronounced stabilization suggests that the formation of this N-C bond is highly favorable. Under continued basic conditions, intermediate **9** undergoes deprotonation at an acidic C–H site with an energy barrier of 29.4 kcal/mol, leading to intermediate **10**, which has a Gibbs free energy of −71.5 kcal/mol. Subsequently, **10** eliminates a hydroxyl anion spontaneously (OH^–^) as the energy barrier is 0.4 kcal/mol only, ultimately yielding the final product **3c**. The Gibbs energy change of the whole reaction is highly exothermic (−101.7 kcal/mol).

From a computational perspective, the above pathway emerges as the most plausible mechanistic route, supported by both kinetic feasibility and thermodynamic stability. The nucleophilic attack and subsequent cyclization proceed with low energy barriers, and the formation of intermediate **9** is highly exergonic followed by a kinetically supported proton transfer, reinforcing its stability. These theoretical insights provide a comprehensive understanding of the reaction mechanism and align well with the experimental observations.

**Fig. 6:**
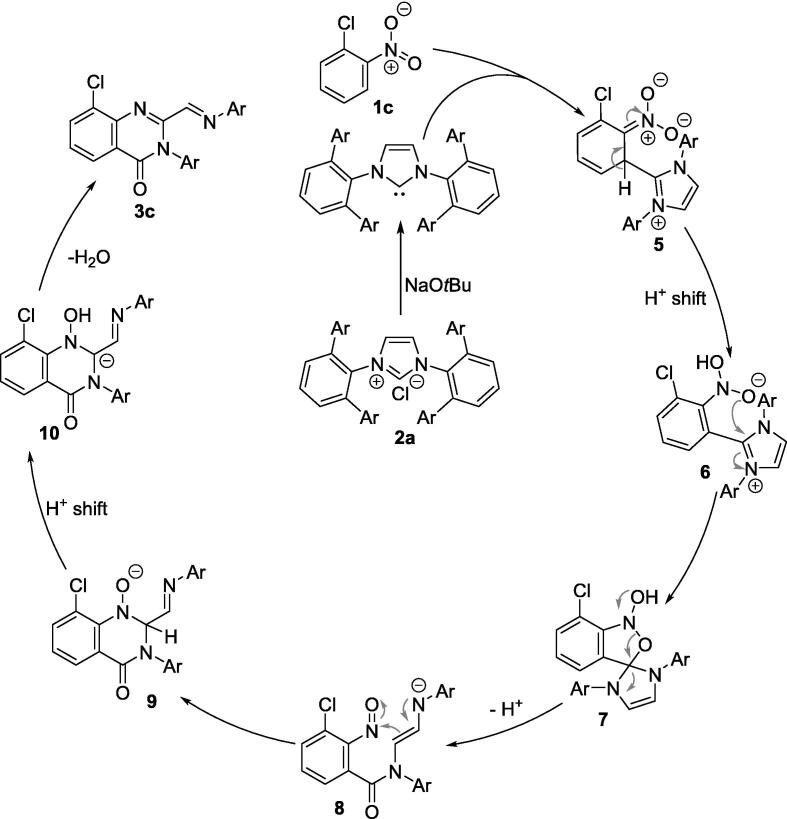
The proposed mechanism. Ar = 2,6-diisopropylphenyl.

Based on the results from both the control experiments and the DFT studies, a postulated reaction mechanism could be obtained as depicted in Fig. 6. The pre-NHC salt **2a** could be deprotonated by the base NaO*t*Bu to generate the free NHC **2a'**, which could add to the *o*-position of the 2-chloronitrobenzene substrate **1a** due to a relatively lower energy barrier than the addition to other positions of **1a**. The adduct **5** that formed from the addition between **1c** and **2a'** could go through an intramolecular proton shift process to rearomatize to the intermediate **6**, which could cyclize through an intramolecular O-Mannich addition to give the spirocyclic intermediate **7**. The intermediate **7** could be deprotonated under basic condition and isomerize to the enamide anion **8** through a ring-opening process, which could be recyclized in the intermediate **9** through the enamide addition reaction. Then the oxide anion in the intermediate **9** could get protonated to give the intermediate **10**, which could be dehydrated to yield the final product **3c**.

### Synthetic transformations of the product **3c**

**Scheme 3:**
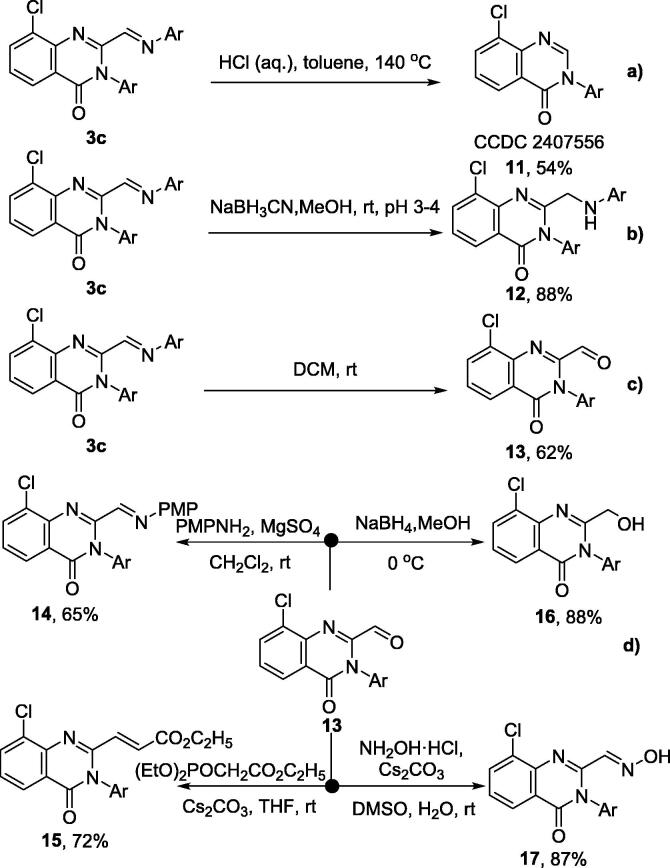
Synthetic transformations of the product 3c. Ar = 2,6-diisopropylphenyl.

The quinazolin-4-one product **3c** contains multiple functional groups and can be readily transformed into various interesting structures with promising synthetic applications (Scheme 3). The *N*-arylimine moiety of the quinazolin-4-one **3c** could be removed to give the product **11** after refluxing in toluene at 140 °C in presence of the aqueous HCl. Reducing **3c** with NaBH_3_CN under weak acidic condition yielded the corresponding amine **12** in 88 % yield. Under mild conditions, the addition of HCl to the dichloromethane solution of **3c** led to the formation of the aldehyde **13**. Condensation of **13** with another amine could produce a new imine product in a moderate yield (e.g., **14**). Additionally, the efficient synthesis of the olefin compound **15** was achieved *via* the Horner-Wadsworth-Emmons (HWE) reaction. The aldehyde group on **13** could also be reduced to generate the hydroxyl product **16** using NaHBH_4_. The reaction of **13** with hydroxylamine afforded the aldehyde oxime **17** in 87 % yield.

### Bioactivity evaluations of the quinazolin-4-one products

Based on the various biological activities of Quinazolin-4-ones, we evaluated the antifungal activities of the synthesized quinazolin-4-one derivatives (detailed activity data for all compounds can be found in the Supporting Information) to identify potential novel leads for pesticides in crop protection. The in vitro antifungal activity was tested using the mycelial growth rate method against four plant pathogens: *Ps* (*Phomopsis* sp.), *Cc* (*Colletotrichum capsica*), *Ss* (*Sclerotinia sclerotiorum*) and *Rs* (*Rhizoctonia solani*) [[57], [58], [59], [60], [61]]. Interestingly, at a concentration of 50 μg/mL, compound **13** exhibited inhibition rates of 58.69 % against *Cc* and 63.16 % against *Ps*, both of which were higher than the commercial pesticide Azoxystrobin. Additionally, compound **13** showed some inhibitory activity against *Ss* and *Rs*, with inhibition rates of 30.99 % and 40.28 %, respectively, slightly lower than Azoxystrobin. (see Table 2) These results demonstrate that compound **13** possesses broad-spectrum antifungal activity, indicating its potential for development as a new scaffold for pesticide design.

**Table 2:** Antifungal activities of the target compounds 13.^a^

Fungi	inhibition ratio (%)	
	Azoxystrobin	13
*Cc*	53.79 ± 1.33	58.69 ± 1.82
*Ps*	45.26 ± 1.57	63.16 ± 1.57
*Ss*	50.70 ± 2.07	30.99 ± 1.86
*Rs*	65.28 ± 1.41	40.28 ± 1.22

a Values of three replicates, with the concentration of 50 μg/mL.

## Conclusions

In summary, we have developed a conceptually novel incorporation reaction between nitrobenzenes and NHC molecules for the facile access to the pharmaceutically significant quinazolin-4-one derivatives. The nitrobenzene could react as a new NHC acceptor and was fabricated with NHC molecules through a complex nucleophilic addition / rearrangement / dehydration cascade process. The reaction mechanism was intensively explored through both experimental and computational studies, which revealed an unprecedented reaction pathway with NHC structures. A diversity of substituents were well tolerated on both the nitrobenzene substrate and the NHC scaffold, with a variety of quinazolin-4-one products afforded in moderate to good yields. The obtained quinazolin-4-one products exhibited promising bioactivities against plant fungi. The NHC and the nitrobenzene were unprecedentedly fabricated through a mechanistically complex while operationally simple reaction. The current featured good atom economy with only one equivalent of water molecule lost. Further investigations into the applications of the newly developed reaction pathway and the bioactive products are in progress in our laboratory.

Compliance with ethics requirements

This article does not contain any studies with human or animal subjects.

## Declaration of competing interest

The authors declare that they have no known competing financial interests or personal relationships that could have appeared to influence the work reported in this paper.
